# Beyond Knowledge and Awareness: Addressing Misconceptions in Ghana’s Preparation towards an Outbreak of Ebola Virus Disease

**DOI:** 10.1371/journal.pone.0149627

**Published:** 2016-02-18

**Authors:** Philip Baba Adongo, Philip Teg-Nefaah Tabong, Emmanuel Asampong, Joana Ansong, Magda Robalo, Richard M. Adanu

**Affiliations:** 1 Department of Social and Behavioural Sciences, School of Public Health, University of Ghana, Legon, Accra, Ghana; 2 World Health Organization Country Office for Ghana, Accra, Ghana; 3 Department of Population, Family and Reproductive Health, School of Public Health, University of Ghana, Legon, Accra, Ghana; University of Oxford, VIETNAM

## Abstract

**Background:**

Ebola Virus Disease (EVD) is not new to the world. However, the West African EVD epidemic which started in 2014 evolved into the largest, most severe and most complex outbreak in the history of the disease. The three most-affected countries faced enormous challenges in stopping the transmission and providing care for all patients. Although Ghana had not recorded any confirmed Ebola case, social factors have been reported to hinder efforts to control the outbreak in the three most affected countries. This qualitative study was designed to explore community knowledge and attitudes about Ebola and its transmission.

**Methods:**

This study was carried out in five of the ten regions in Ghana. Twenty-five focus group discussions (N = 235) and 40 in-depth interviews were conducted across the five regions with community members, stakeholders and opinion leaders. The interviews were recorded digitally and transcribed verbatim. Framework analysis was adopted in the analysis of the data using Nvivo 10.

**Results:**

The results showed a high level of awareness and knowledge about Ebola. The study further showed that knowledge on how to identify suspected cases of Ebola was also high among respondents. However, there was a firm belief that Ebola was a spiritual condition and could also be transmitted through air, mosquito bites and houseflies. These misconceptions resulted in perceptions of stigma and discrimination towards people who may get Ebola or work with Ebola patients.

**Conclusion:**

We conclude that although knowledge and awareness about Ebola is high among Ghanaians who participated in the study, there are still misconceptions about the disease. The study recommends that health education on Ebola disease should move beyond creating awareness to targeting the identified misconceptions to improve future containment efforts.

## Introduction

Ebola Virus Disease (EVD) is a severe, often fatal illness, with high mortality rates. The case fatality rate of the West African EVD epidemic which started in 2014 has been reported to be 70% [1,2]. The illness can affect both humans and non-human primates (monkeys, gorillas, and chimpanzees). The origin of the virus is unknown, however available evidence suggests that fruit bats (Pteropodidae), apes and chimpanzee are the likely host of the virus [3]. The incubation period is between 2–21 days, with an observed average of 8 to 10 days [4,5].

EVD is a zoonotic infection and historically has been reported to have initially been transmitted from animals to human beings. However, infections can also occur between humans through direct contact with broken skin or mucous membranes, or other bodily fluids or secretions [3]. The infectious virus and/or viral RNA has been reported to be present in bodily fluids such as blood, sweat, stool, urine, saliva, semen and breastmilk of infected people [3,4]. Infection can also occur if the broken skin or mucous membranes of a healthy person comes into contact with environments and items that have become contaminated with an EVD patient’s infectious fluids such as soiled clothing, bed linen, or used needles [3]. In Liberia, motorbike-taxis have been reported to be a very important source of potential EVD transmission, as these vehicles were often not disinfected after carrying people with EVD to treatment centres before new passengers are taken on board [6]. There is also evidence that EVD virus can be viable in semen up to ninety days after clinical recovery from the illness [4]. Recently, the possibility of sexual transmission has also been reported [7–9].

A person infected with EVD may not be contagious until symptoms appear. Symptomatic patients therefore pose a greater risk to others. EVD may present with different signs and symptoms ranging from non-specific manifestations to haemorrhagic ones. The nonspecific manifestations mimic common tropical illnesses like malaria and typhoid fever with sudden onset of fever, chills, myalgia (muscle pain), and malaise. Patients may also show flu-like symptoms such as nasal discharge, cough, and shortness of breath. Infected persons may also present gastrointestinal symptoms such as diarrhea, nausea, vomiting, and abdominal pain. Hemorrhagic symptoms may also manifest in the most severe cases [3,4]. Hemorrhagic manifestation may be overt, subtle or mild in which case the manifestations may often be missed. Overt hemorrhagic manifestations occur in the terminal phase of the illness and are preceded by multiple organ failure. A review of the case series in the West African epidemic that started in 2014 has showed that the most common symptoms reported between onset and when cases were detected included: fever, fatigue, loss of appetite, vomiting, diarrhea and headache [1]. Haemorrhagic manifestation were reported in less than 6% of cases [1]. Death from EVD occurs as a result of severe and uncorrected electrolyte imbalance [3]. Nonetheless, some patients survive and recover from the condition.

The West African EVD epidemic that began in 2014 has had a negative impact globally. Although the outbreak originated in rural Guinea, it spread to Liberia and Sierra Leone reaching urban areas in these two countries, a factor that distinguishes this outbreak from previous episodes which had remained rural [4]. This epidemic received much international attention and was declared a public health emergency of international concern by the World Health Organization. As of November, 22 2015, a total of 28,601 confirmed, probable and suspected cases of EVD have been reported in eight affected countries (Guinea, Liberia, Mali, Sierra Leone, Nigeria, Senegal, Spain and the United States of America) with 11,299 deaths [10]. During this outbreak, people who were at greater risk included health workers, family members or people in close contact with infected individuals; and mourners who had direct contacts with the bodies of the deceased as part of burial ceremonies [5]

Knowledge on the epidemiology of EVD is very important in any EVD prevention and control intervention programme. A recent study in Sierra Leone reported that majority of the respondents were aware of the existence of an EVD outbreak in the country. Also, about 77% of 1,413 people interviewed said they had heard of someone who survived EVD [11]. The study also identified some misconceptions, as a third of respondents believed that EVD was a result of sufferer’s sins. Furthermore, about a quarter of respondents believed EVD could be spread through mosquito bites [12]. A similar study in Lagos, Nigeria, also reported high level of knowledge on EVD with about 92% (304/331) and 84% (278/331) of respondents being aware of the direct and indirect modes of transmission respectively [12]. Contrary to this finding, another study in Nigeria reported that only 41% of the respondents possessed satisfactory general knowledge about EVD whilst 44% and 43.1% of 5,322 respondents were reported to possess satisfactory knowledge on mode of spread and preventive measures, respectively [13]. Though both studies were conducted in Lagos, Nigeria, the former had a relatively small sample size and was conducted in 10 local government areas whilst the latter was conducted in 12 out of the 20 local government areas and with a more diverse sample size. These studies were also conducted at difference times during the EVD outbreak in Nigeria. Knowledge and level of awareness may often change over time during an outbreak as health education is intensified. Therefore, these two reasons could be responsible for the observed differences in the level of knowledge among the respondents in these studies. Nonetheless, misconceptions were reported in Nigeria where nearly one‐third of respondents held the belief that EVD could be contracted through air or through mosquito bites while one-fifth believed that spiritual healers could successfully treat the disease [12]. Furthermore, misconceptions were reported among health workers as 15% (18/117) of respondents and 1 in 10 believed EVD could be contracted through the air and through mosquito bites, respectively [12]. In a related study among Australian Hajj Pilgrims, a majority (134/150) of respondents reported that they were aware of the epidemic before travelling, and another 60% (80/134) were aware that EVD was transmitted through body fluids [14].

Ghana had not had any confirmed EVD case though the high trans-border activities between Ghana and other affected West Africa countries placed Ghana at risk [15]. In preparation to handle any EVD outbreak, Ghana established an inter-ministerial committee under the leadership of the Minister of Health and formally developed a national EVD preparedness and response plan in August 2014. This plan is structured around five thematic areas: planning and coordination; surveillance, situation monitoring and assessment; case management; social mobilization and risk communication; and logistics, security and financial resources [16]. In addition, EVD response teams have been established at national, regional and district levels. To equip stakeholders with knowledge on how to manage EVD cases, training of frontline health workers were conducted across Ghana. Furthermore, national and regional EVD treatment centres were established [16] and Personnel Protection Equipment (PPE) supplied to health facilities. As a measure to prevent the importing of the disease and to ensure early detection of EVD among international travellers, screening exercises were undertaken at various entry and exit points in Ghana. There was also been an increase in health education and community mobilization efforts in preparation towards containment in the event of an outbreak. This study therefore explored community knowledge and awareness about EVD and its transmission and the role misconceptions can play in Ghana’s ability to contain an outbreak.

## Materials and Methods

### Ethics Statement

The study protocol was reviewed and approved by the Institutional Review Board of Noguchi Memorial Institute for Medical Research in Ghana with reference number NMIMR-IRB CPN 057/14-15. This institute is mandated to review biomedical research protocol in Ghana and have Federal-wide acceptance. Informed written consents were also obtained from participants and each participant was identified using a unique number instead of their names to ensure anonymity and confidentiality.

### Study Design

The study design was descriptive adopting a qualitative approach to data collection. A qualitative research approach was deemed appropriate to gain deeper insight into knowledge and perceptions about EVD from the perspectives of community members and health care providers. The study combined narrative and phenomenology strategies in qualitative enquiry. Narrative research allows participants in a study to share their experiences in the community [17]. Phenomenology on the other hand allows participants’ to share their perceptions, feelings, and lived experiences in the community and how these experiences may affect their perspectives about a given situation [18].

### Study Area

The study was conducted in the Republic of Ghana. Ghana is located on West Africa's Gulf of Guinea only a few degrees north of the Equator. Ghana shares borders with Côte d'Ivoire to the west, Togo to the east, and Burkina Faso to the north. Ghana is also bordered to the south by the Gulf of Guinea and the Atlantic Ocean. The country has a varied geography of savannas, forests, woodlands, mountainous areas, and coastal borders. The population of Ghana is estimated as 24,658,823 with an annual growth rate of 2.4 percent [19]. The nation has ten administrative regions, which are further divided into metropolitan, municipality, districts and sub-districts. There are about 75 ethnic languages spoken in Ghana with English as the national language of communication.

This study was carried out in five of Ghana’s ten regions with a combined population of 15,764,171. The five regions were Greater Accra, Western, Volta, Ashanti and Northern. The Greater Accra region (GAR) and Ashanti region (AR) are the two most populous regions in Ghana with populations of 4,010,054 and 4,780,380 respectively, and corresponding population densities of 1,236 and 196 per square km [19]. The Volta Region (VR) has a population of 2,118,255 whilst the Western Region (WR) has a population of 2,376,021 [19]. Northern Region (NR) has a population of 2,479,461 [19] and is the largest among the three regions in the northern part of Ghana.

The selection of these regions were based on rural-urban dynamics, population density and entry and exit points. The Greater Accra and Ashanti regions were selected based on their high population densities. High population density has been noted to increase both animal-to-human and human-to-human EVD transmission [20]. Also, the Greater Accra region is the main entry point for sea and air travels. The Volta Region shares borders with Togo at Aflao whilst the Western Region shares borders with Cote D’ Ivoire at Elubo and also serves as major sea port. The two Regions (Volta and Western) were therefore selected because they serve as entry and exit points in Ghana. The Northern Region also shares borders with Cote D’Ivoire to the west of Bole District.

### Data Collection Methods

#### Focus Group Discussions

Twenty-five Focus Group Discussions (FGDs) were held in this study; five in each of the five regions. Two FGDs were conducted among male community member, two among female community members and one amongst nurses. Each region was stratified into urban and rural and one FGDs held for males and females making a total of 10 FGDs in rural and 10 FGD in urban areas as shown in (Table 1). FGDs with nurses were not stratified by sex as both male and female nurses were put together for the discussion. Each group was made up of between 8–10 individuals with a total of 235 participants in all. The discussants were made to sit in a semi-circular fashion with the moderator and note-taker in the middle. During FGDs, each participant was given the opportunity to contribute to any question asked before moving to another question. Generally, it took between 60–90 minutes to complete a FGD.

**Table 1:** Summary of FGDs composition.

Region	Females		Males		Nurses
	Rural Community	Urban community	Rural community	Urban community	
Greater Accra	Ada	Kanda	Asutuare Congo	Tema	Accra
Western	Sefwi Daatotano	Elubo	Nkwantakese	Bibiani	Takoradi
Ashanti	Amanchia	Konongo	Kusa	Offinso	Kumasi
Northern	Kawsawgu	Walewale	Malzeri	Damongo	Tamale
Volta	Klefe	Tsito	Fume	Aflao	Ho
Total	5	5	5	5	5

#### In-depth Interviews

Forty (40) In-Depth Interviews (IDIs) were carried out, eight in each of the five regions. The participants for the IDIs were drawn from both rural and urban communities in the study areas. These participants included male and a female community members, an immigration officer, a chief or any other opinion leader, two health workers and two members of the EVD response task force members. In Western, Ashanti and Greater Accra Regions with ports, one port health official was further interviewed in these regions as shown in (Table 2).

**Table 2:** Summary of IDI Participants.

Professional Background/Position	Rural	Urban	Total
EVD Response Team Members	-	10	10
Immigration Officers	1	4	5
Port Health Officials	-	3	3
Chiefs	3	3	6
Religious Leader	1	-	1
Assemblymen/Assemblywomen	2	3	5
Health Workers	-	10	10
Total	7	33	40

### Selection of Participants

The study included males and females who were adults aged 18 years and above as required for informed consent in Ghana [21]. Purposive sampling technique was employed in selecting participants for both FGDs and IDIs. Each region was divided into two strata (rural and urban) for the purposes of this study and participants were recruited from each of these strata for the FGDs and IDIs. Community leaders in these areas were also purposely selected and interviewed as key informants. In addition, health care providers, immigration officers and two EVD task force members in each of these regions were purposely recruited and interviewed to provide expert views on their EVD prevention and control strategies. Both male and female professional nurses who are frontline staff in health facilities were also purposely selected for FGDs in each of the regions. The age range of male participants was between 20 to 76 years whilst female participants ranged between 20 to 86 years. The highest educational attainment of participants was tertiary education and these were mostly participants from urban areas. However, some of the participants in rural areas also had formal education and were mostly civil servants in those communities. The participants adhered to three main religious affiliations: Christianity, Islam and African Traditional Religion. Some of the respondents were married whilst others were single, either never married, or had lost their partner. A participant’s number of children ranged from no children to nine children.

### Data Collection Tools

Semi-structured IDIs and FGDs guides were the main data collection methods used in the study. These guides were designed in English and translated to the local languages by language experts using a back-to-back strategy. In this strategy, a language expert proficient in English and any of the local languages was made to first translate the interview guide from English language to the local language. Another language expert was then made to retranslate the guide from the local language back to English and the two versions compared. Where there were differing views, these were discussed by the two language experts with a third language expert as a mediator. This was done as a quality control measure to ensure uniformity in the data collection tools. The FGDs guide covered areas such as knowledge on causes and modes of transmission, signs and symptoms as well as attitude about EVD. In addition, the FGDs guides explored areas such as local beliefs about EVD, and its modes of transmission. IDIs guides also explored areas on knowledge, preparation towards containment, screening at various entry points and community mobilization strategies. Fifteen graduate research assistants were recruited, trained, put into teams and deployed to their linguistically competent regions to collect the data from the 10^th^ of February, 2015 to the 3^rd^ of March 2015. There were three members in each team.

### Data Analysis

All FGDs and IDIs were digitally recorded with the participants’ permission. Field notes were also transformed into data documents within a day of the IDIs or FGDs. These notes, as well as transcriptions, were anonymised, and no identifying information was included in the notes. Each participant was assigned a unique number instead of using their names. Data collected in the local languages were first translated into English by two independent people. The translations were then compared for consistency. Any inconsistency was discussed by the translators with a third person serving as a mediator. All transcripts were again reviewed by an independent person. In the review, the independent person listened to the various recorded voices and compared the voices with the transcriptions. Qualitative narrative data in English were then entered into a word processor (Microsoft Word) and imported in a format that allows coding of the interview transcripts in Nvivo 10. Double coding was done for each transcript and compared.

This study was to explore community knowledge about EVD with the aim of identifying gaps and developing strategies to address these gaps in the event of possible outbreak. For this reason we adopted a framework analysis [22]. This approach allowed us to include the model in data analysis, and allowed emerging new data to inform the model at the same time. The steps in framework analysis include; familiarisation, identifying a thematic framework, indexing, charting, mapping and interpretation.

Though there are distinct steps in the analysis, the process itself is iterative, going back and forth between the different steps, ensuring that the analysis is grounded in the data [22]. To start with, three analysts read and re-read the transcripts, to become familiar with the content. Thereafter, a codebook was developed by the three analysts, discussed and accepted. The codebooks contained the various codes that are to be used in coding the data, the definitions of the codes, examples of situations where such codes should be used as well as examples of instances where it is inappropriate to use such codes. This was then used to code the data, as well as examining the relationship between the codes for the purpose of creating categories and matrix. An independent analyst checked the codes, relationships, categories and matrix against the original transcripts to ensure uniformity. The analysts then went back to the transcripts, after changes have been made to the matrix, and coded transcripts according to the coding matrix. As coding continued, categories were developed, which then evolved into themes.

The last two steps have also been described as developing descriptive and explanatory accounts of the data [23]. To develop a descriptive account of the data, the researchers wrote a description of the range of codes, categories and themes emerging from the data by constantly referring to the original transcripts, to ensure accuracy. The explanatory accounts then involved making sense of the descriptive accounts, and interpreting findings in the context of existing literature. To facilitate this, the data were stratified into attributes such as gender, region, rural and urban and each attribute was linked to the nodes and data sources. Queries were run on various nodes against attributes to compare and contrast within-group and between-group responses. The data were than explored using the attributes and nodes to draw frameworks capturing various aspects of the data.

## Results

### Knowledge on modes of transmission of Ebola Virus Disease

The study generally revealed a high level of awareness about EVD. Also, knowledge about EVD particularly how it is transmitted to humans was high. Respondents were able to identify the major forms of transmission, that is, animal-to-human and human-to-human modes of transmission. With human-to human mode transmission, respondents knew about both direct and indirect transmission. Respondents mentioned that coming into contact with bodily fluids of infected persons as a direct mode of transmitting the disease. They also said transmission occurs when one eats an infected animal, and/or washes and touches dead bodies of relatives infected with the disease. These findings permeated both rural and urban settings across the regions as evident by the quotes from respondents:

“If an animal or human being die from Ebola and you touch the animal or human being without protection, you can get the condition” (FGD, Females, Rural, AR).

“Ebola is present in body fluids like the blood, saliva and water from the body, if you touch water from the infected person you will get it” (FGD, Male, Urban, AR).

“They have made us understand that if someone has Ebola and the person’s sweat touches you or you shake the person you will get the disease so it is not just through eating meat that you can get the disease” (FGD, Female, Rural, GAR).

“What I want to say is that I heard on radio that if someone has the disease and you have body contact with the person you can be infected, or if you shake the person you can get Ebola, so we should stop shaking hands” (FGD, Male, Rural, GAR).

“If someone has it and you go near the person or greet, hand shake him or her one can be infected or exchange of fluids from an infected person example is sweat, blood” (FGD, Female, Urban, VR).

“If an Ebola patient dies and you go and carry the body trying to place it well you can be infected with the disease and spread it” (FGD, Female, Urban, WR).

With regards to indirect mode of transmission, respondents reported that one could get infected with Ebola through touching the clothing of infected persons, and any other items such as bed linen, cups, and bowls, car seats contaminated with body fluids from someone who has the disease or has died from it. The following responses illustrate the respondents’ knowledge about indirect transmission:

“…it is not only coming into contact with the infected persons body fluid but also personal belongings such as clothes that are infected” (FGD, Nurses, GAR).

“If Ebola kills somebody and you touch the dead body without protective clothing, you can also get the disease” (FGD, Male, Urban, NR).

Whereas respondents’ knowledge of direct modes of transmission emerged spontaneously from the interviews, the indirect ways of getting transmission were solicited through prompting by moderators particularly in FGDs in many instances. Across the regions, the direct ways of acquiring Ebola remained better known than the indirect ways. In terms of knowledge and awareness, the study did not show much difference across various regions.

Many respondents believed that an individual infected with Ebola even without the onset of signs and symptoms was capable of infecting others. In FGDs across the regions the same views were held regarding asymptomatic transmission as illustrated in this discussion.

“….Though the person is not showing any signs and symptoms, it will be in the blood, and can be transmitted” (FGD, Female, Rural, AR).

“If you touch the body fluids of that person you can get the disease like if you touch the sweat of that person you can get the disease” (FGD, Female, Rural, NR).

“Yes it is possible for someone like that [asymptomatic] to infect others… once the person has the virus inside the body, people have different blood groups and immune systems so someone may have it but not show symptoms, so it’s possible that person can infect others” (FGD, Male, Rural, GAR).

Evidence from the study revealed that health workers also held a firm belief that people who did not manifest the signs and symptoms could transmit Ebola as illustrated by the health workers below:

“It will depend but I will say yes because once the organism is in the system, the person can still transmit it to other people. Because like I was saying if he has resistance and the immunity is very good, the signs will not show and it will be in his [her] system and if you come in contact with the person, you can get it. Such a person will be a carrier which means the person has the virus but has not develop the condition but can transmit it to other people” (IDI, Health Worker, WR).

“Yes you can pass it on to other people as long as the virus is in your system” (FGD, Male, Nurse, VR).

“Yes, you can pass it on because the disease is within you and if you touch anybody or anybody touches you, you can pass it on to them (FGD, Female, Nurse, NR).

Nonetheless, some of the health workers also reported that people who did not show signs of the disease were incapable of infecting others as mentioned in the quote below:

“It’s believed or it’s been documented that the person is not infectious until he [she] starts showing signs and symptoms” (FGD, Nurse, GAR).

### Knowledge on incubation period, signs and symptoms

Regarding the incubation period for Ebola, results from the study showed that respondents generally knew how long it will take for an affected persons to start to manifest sign and symptoms. Respondents also identified that the incubation period could depend on the immune system of the individual. Generally, respondents mentioned between 2 to 21 days as the incubation period as observed by respondents:

“It takes between 2 to 21 days from the point of infection to start showing signs and symptoms” (FGD, Female, Urban, WR).

“If I remember correctly, it is about 21 days and within the 21 days depending on your body immunity level, it may take some few days before it shows” (IDI, Paramount Chief, NR).

The study further explored respondents’ knowledge on non-specific signs and symptoms of the disease. Findings revealed that community knowledge on non-specific manifestations such as headache, fever and chills was high. However, many wondered how these signs could be differentiated from conditions such as malaria and typhoid fever which respondents perceived to be common conditions in the community. Gastro-intestinal manifestations were also widely known by respondents and were mentioned spontaneously without prompts in this study across all the regions as observed in the FGDs.

“We have not had a case in this community but we [have] heard some of the symptoms include vomiting, diarrhea, cold, and headache. However, malaria has similar symptoms so we can only confirm it is Ebola if the person is taken to the hospital” (FGD, Male, Urban, NR).

“The signs of Ebola include headache and the person will also run temperature (Fever) and you have running nose” (FGD, Male, Rural, GAR).

“Suspected Ebola case will have loose stools, will be vomiting and also have fever, so when you see though things, you need to suspect the person as a possible Ebola case” (FGD, Male, Rural, AR).

Furthermore, haemorrhagic signs and symptoms were widely mentioned in FGDs in all the regions that took part in this study. These were generally believed to be the only typical signs of Ebola as the onset of haemorrhagic symptom was perceived as signs of a worsening and terminal condition:

“If I see that the person is weak and blood is coming out of the person’s ears and nose and the person is vomiting and has difficulty in breathing, then I will know that the person has Ebola, but for the bleeding, once you see that it means death is approaching” (FGD, Male, Rural, AR).

“If the disease attacks, there will be severe headache over a prolong time amidst vomiting and passing of watery stools. The patient becomes generally weak. If the patient delays at home, blood starts oozing from all possible “holes” (orifices) of the person. Death is inevitable even if he/she is rushed to the hospital” (FGD, Female, Rural, WR).

### Misconceptions about Ebola Virus Disease

Despite the high knowledge demonstrated by respondents, there were still some misconceptions among community members. For example respondents attributed the Ebola outbreak in some countries as a punishment for their sins against God. In their opinion, Liberia and Sierra Leone had Ebola outbreaks because of the atrocities committed during civil wars in those countries. Respondents believed that the wars resulted in prolonged famine. As a result, people were compelled to consume human flesh to survive. Therefore, these countries are being punished with this disease because people consumed human flesh in the past which is a sin. This way of thinking emerged in both FGDs and IDIs throughout Ghana:

“Yeeh I heard…a friend told me that because in Liberia people ate dead bodies of their colleagues because of the war, so that is why they are getting Ebola” (FGD, Nurses, GAR).

“….Recently, a woman told me that, it is because of the way people were killed in Liberia, Sierra Leone that is the reason why God is angry with them and has cursed them. So, we should be prayerful in Ghana for God to forgive us. She continued that wars are not good, so we should pray against it otherwise Ghana may also be cursed” (Health Worker, IDI, NR).

Another misconception about Ebola identified in this study was the belief that Ebola is airborne. This belief was deeply rooted in the way health care workers taking care of Ebola patients dress on television. Respondents were convinced that the only reason why people caring for Ebola patients cover all parts of their body including their noses and faces was because Ebola could be transmitted through the air.

“Yes, we heard one can get the virus through the air. If you interact with an infected person, they may pass it to you through coughing and sneezing. That is the reason why they [care providers] dress like that on television, covering all parts of their body” (FGD, Male, Urban, NR).

“The disease is in the air hence when he [infected person] speaks to me I will become infected, I will get it” (FGD, Females, Rural, VR).

“…. I also heard that a doctor died of Ebola, what I asked myself is, this doctor protected himself before attending to the Ebola patient so if Ebola is not in the air how can the doctor just get close to the patient and contract the disease which affected him and he did. So I asked and I was told that it is through the air the doctor got it” (FGD, Women, Rural, GAR).

This believe that Ebola could be transmitted through the air was also common among health care providers. Although health workers who had received training on ebola were informed that Ebola could not be transmitted via air, respondents still had some doubts about this claim as expressed by health workers below:

“…and maybe if an infected person sneezes or coughs and the droplets comes out and I happen to be close by and through the air I breathe in I can also get it. That is the reason why health worker get infected” (FGD, Female, Nurse, AR).

“I am trained, but during our training we were told that one could not get Ebola through the air but I don’t believe that because some of the experts in the use of PPE still got the condition. So I think they could have only been infected through the air” (FGD, Female, Nurse, GAR).

Another misconception reported by respondents was the ability of mosquitoes and houseflies to transmit Ebola from infected persons to non-infected individuals. Some respondents reported that mosquitoes can take the disease from an infected person’s blood to another person. Houseflies on the other hand were believed to be capable of transmitting the infection through contaminating food or through the release of contaminants on the skin of people.

“As for the mosquitoes, it is even serious because, they will suck the blood of the infected person, take some of the blood, and when they bite you to suck your blood, then it will leave some of the organisms in your blood. It is just like how the mosquito give people malaria by biting people with malaria in their blood and put the malaria in another person’s blood to get the condition” (FGD, Female, Rural NR).

“I agree with what number 5 has said, If someone has it and sweats, and the houseflies settles on the sweat, it can pick some of the disease and when it settles on your body too, then it will leave the condition on your skin or if it is your food, then you get the condition” (FGD, Male, Urban, VR).

“I am very sure it [mosquito] can give it (transmit) because if it bites and sucks your blood which is infected and injects it into another person that person can contract Ebola” (FGD, Male, Urban, WR).

### Misconceptions about Ebola Virus Disease, stigma and discrimination

Findings from the study revealed that misconceptions could result in stigmatization and discrimination. The following responses show the extent to which community may stigmatize a suspected EVD patient, as discussants had this to say:

“….Those who are staying with the person at home will be afraid because even flies and mosquitoes can infect you. So I can say that the whole community will be afraid. Nobody will be willing even to come to the house in which the person stays” (FGD, Female, Rural, AR).

“…precisely, we are talking about Ebola if my wife is infected and I take the fact that oh darling then I touch you and get infected what about the children, at least one of us should live to take care of the children, I will leave with my children until she recovers because if we stay with her the flies will infect us” (FGD, Male, Urban, GAR).

Stigma and discrimination against Ebola cases is also likely to extend to the household and other family members as well as people staying in the same neighbourhood with the Ebola patient. In response to a question on what respondents will do to a suspected case of Ebola, a respondent in FGD in Northern Region had this say:

“I will not go closer at all. I will be scared to even interact with members of his/her family because, he/she might have given it to the family members before it was diagnosed (FGD, Male, Rural, NR).

Furthermore, the study revealed a possible stigmatization of even health workers who will agree to work in Ebola treatment centres based on these misconceptions. In a FGD with nurses, respondents were generally of the view that any of their colleagues who agree to work in Ebola treatment centre will be isolated and stigmatized as illustrated:

“For the period… we can talk on phone but not my house” (FGD, Female, Nurse, GAR).

“Not my house, not even my backyard” (FGD, male, Nurse, AR).

“I will set a rule and say it seems now you are chasing money so our friendship is a phone friendship, don’t come to my house” (FGD, Female, Nurse, WR)

“Ebola is like another name for death, so you see death coming and you embrace it” (FGD, Female, Nurse, NR).

The stigma for suspected cases will not only occur when the person has the condition but will also continue after the person has been treated and has recovered from the condition. Some people will still not be comfortable interacting with people who have been confirmed as Ebola patients despite being declared cured. In a response to a question on the likely attitude of discussants to a person who has recovered from Ebola, a male participants in an FGD organized among rural residents in Western Region stated:

“A dead snake is still fearful- fear is there I will not come so close” (FGD, Male, Rural, WR).

“I won’t go near him [her] at all because if a mad man is healed from madness, he [she] still has a little of madness to scare kids” (FGD, Male, Rural, WR).

The likelihood of post-EVD stigma was widespread across the regions in Ghana and may even be carried over to other social gatherings. Respondents observed that once the person has had EVD, nobody would like to interact with such a person in any social gathering.

“If a person is a Muslim and join us in congregation, people may desert the line on which the victim stands. They may get no one to stand with. They must remain at home until we finally believe that we will no longer get it from them” (FGD, Female, Rural, NR).

The study also revealed that health workers were also very apprehensive of dealing with suspected Ebola patients. Interviews with health workers and FGDs with nurses also revealed the possibility of similar attitudes towards suspected cases of Ebola. The misconception of linking how other health workers acquired the disease whilst using PPE to airborne nature of Ebola made health workers apprehensive as illustrated:

“It doesn’t matter how well I am trained or paid, even the Americans they came to train the people but they got it, so [as for] me I won’t take that risk” (FGD, Female, Nurse, AR).

“One time there was an incident at the A&E (accident and emergency), a taxi driver brought in a patient and then the nurses triaged the patient alright and then after a while it was like most people have gotten in contact with the person and then at a particular point they noticed it was a suspected Ebola case and then everybody started running here and there” (FGD, Female, Nurses AR).

As a result of the stigmatization against Ebola patients and their households, some households may prefer to hide suspected cases as illustrated by a nurse in Western region.

“…the perception about [name of community withheld] community is very deadly. Most of the people are fisherman, most of them travel to Liberia and those places. So when they have relatives with high temperature, they are prepared to hide the person. So that the person is not isolated. We have about four suspected cases but their relatives are not ready to show them up” (FGD, Nurses, WR).

Another area of concern is how these misconceptions could affect the health seeking behaviour of community members. Respondents in rural Ashanti region had a strong belief that Ebola was a spiritual disease and as such could only be prevented through prayers and divine intervention. Also, in rural Northern region, respondents believed that Ebola was caused by witchcraft. It was also reported that, one could be infected with Ebola if you eat food prepared by an evil person. They also thought that offending a witch could lead to being inflicted with EVD. Though these were mentioned in all regions in the study, it was well-entrenched in the Northern Region. It is also worth noting that a health worker (a nurse) also believed in spiritual cause of the disease. The responses below illustrates the argument above:

“Ebola is spiritual condition, hence when we go on our knees to pray, we pray for forgiveness and ask God that He should not allow this Ebola to get into our community” (FGD, Rural, Female, AR).

“It is a spiritual disease and even the Bible attest to the fact that a time will come when there will be a disease and there will be no cure for it and it is killing people” (FGD, Rural, Female, AR).

“… Ebola can be caused through witchcraft, they say Ebola can be caused through eating from your enemies pot, something like that I have ever heard of such beliefs” (FGD, Male, Nurse, NR).

Based on these perceptions, respondents mentioned that it will be important to seek care for Ebola from spiritual sources that has the means to deal with such powers and not formal hospitals.

“If you get it through witchcraft, then you need deliverance and spiritual healing from someone with such powers. There are several of such people in our communities” (FGD, Female, Rural, NR).

“Spiritual condition like Ebola are better handle by spiritual healer, so it would be better to go to such place rather than the hospital. So you see that even in the places with Ebola, many of people who are sent to hospitals die. This is because it is spiritual” (FGD, Male, Rural, NR)

## Discussion

### Knowledge about signs and symptoms, causes, prevention of Ebola Virus Disease

The general awareness about EVD was high and every participant in this study had heard about the disease. Specific knowledge on modes of transmission, and clinical manifestations were also high. Animal-to-human transmission was mentioned spontaneously in all FGDs and IDIs. Direct transmission through human-human contact and contacts with body fluids of infected individuals was also widely known and emerged as an unprompted response. Other studies in Nigeria and Sierra Leone have also reported high knowledge on EVD with more than 70% of respondents in each of those studies able to identify both direct and indirect human-to-human modes of transmission as well as animal-to-human transmission [11,12]. However, another study in Nigeria reported a lower level of knowledge as only 41% of the 5,322 respondents possessed satisfactory general knowledge with 44% possessing satisfactory knowledge on mode of spread of EVD whilst 43.1% possessed satisfactory knowledge of preventive measures. Knowledge on modes of transmission is important as EVD can be prevented using simple measures such as observance of diligent hygiene practices. Hand washing has been recommended as essential at homes, work places, health care centers and Ebola care centres for preventing the spread of EVD [3].

As knowledge and awareness of EVD is high among respondents, it is important for health education to now focus on why people need to change the high risk behavioral practice that are known to influence containment. Findings from this study revealed that respondents clearly identified the major behavioral practices such as touching of infected people, touching of items contaminated with bodily fluids, and eating of infected animals. As observed in several settings, knowledge of high risk behavioral practices that are imbibed in everyday life does not necessarily translate to positive behavioral practices change [24–26]. Studies have also showed that communicating knowledge about why people should wash their hands with soap, sleep under a bednet, or change their sexual practices is often insufficient to induce behavioural changes in practice especially in situations where alternative health priorities exist [27,28]. To induce positive behavioural change will require addressing community concerns about these practices through constant engagement at all levels to influence adoption of new behaviours.

Although respondents in this study were able to identify the early signs and symptoms of EVD, they were still faced with a challenge as to how to clearly distinguish it from other illnesses such as malaria, typhoid fever, gastrointestinal infections and respiratory tract infections that present similar signs and symptoms as EVD. It is important to note that respondents may misconstrue the early signs and symptoms of EVD for these endemic conditions. In Uganda for example, during the year 2000 Ebola haemorrhagic Fever outbreak, it was reported that at first, many persons treated the symptoms of Ebola Haemorrhagic Fever as a regular illness and sought a variety of both biomedical (malarial drugs or antibiotics) and indigenous cures (herbs, traditional healers) [29]. It is therefore important to emphasize these similarities in health education and people should be advised during outbreaks to seek treatment at biomedical facility irrespective of what they perceive to be the cause of their illness.

### Addressing misconceptions towards Ebola Virus Disease preparedness and containment

Although this study reported that knowledge on EVD was high across the country, several misconceptions about EVD were also identified. These misconceptions are categorized into two main sub-themes: misconceptions regarding the modes of transmission and misconceptions concerning the management of EVD. The belief that EVD was spiritual, caused by witchcraft or supernatural conditions has implications for the prevention and care of EVD patients. This belief in supernatural causes will lead individuals to seek spiritual remedies in either churches or other spiritual outlets without going to the hospital. Several studies have reported that when disease conditions are attributed to spiritual causes, people seek help in non-orthodox health outlets as biomedical facilities are believed to be ineffective in handling spiritual or supernatural conditions [30–32]. This perception has the potential to change sources of seeking health care for EVD from orthodox health facilities to traditional or folk centres and as such serve as potential exposure grounds. Involving people who operate these healing centres and the entire community leaders could assist in ensuring that suspected cases are not left at these healing centres to infect other people. In times of national emergencies like an EVD outbreak, it is very important to identify such alternative health care sources within each community and position trained health workers to screen clients for EVD infection.

This study reported that respondents believed that EVD could be transmitted through the air and this misconception was supported by what most people saw daily on their television sets. This misconception emanated particularly from the PPEs health workers wore to provide treatment for the EVD patients. For example, PPEs created fear and anxiety and reinforced the idea that the disease could be transmitted through air because the PPEs were seen as airtight. It has been observed that images provided as a sources of information may shape attitude and influence behaviour [33,34]. Information provided to the public that turn out to be unfavourable, misinterpreted or scary could contribute to stigma and discrimination and present a challenging barrier to treatment and prevention of disease [33–37]. In a study in Sierra Leone, nearly one-third of households believed that EVD was transmissible through the air or mosquito bites [11]. This study also revealed that communities perceived EVD to be an airborne condition. There is therefore the tendency that suspected EVD cases could be neglected because of fear of getting infected through the air. Health education needs to focus on this perception as studies have showed that EVD transmission through the air is rare and absent in natural outbreaks [38], but has only been identified in laboratory settings [39]. This misconception could also lead to over estimation of the risk of contracting EVD through houseflies, mosquitoes, and through the air. Overestimation have been reported to be associated with discriminatory attitude to people with infectious diseases [40,41]

A table summarizing the findings of the study and how misconceptions could possibly affect the prevention and control of EVD (Table 3).

**Table 3:** Interactive effect of misconceptions on EVD control.

Misconceptions	Outcome	Effects	What to do
Misconception related to perceived causes include EVD is a:	The misconceptions on the perceived will result in people seeking for health care from:	Containment challenges	What can be done to address contain situation:
Spiritual condition	Spiritualists	Containment challenges	Identifying non-orthodox health care providers in communities and training them on EVD
Punishment by God	Faith Healers	Containment challenges	Using non-orthodox health care providers to identify cases that report to them and aid in contact tracing
Witchcraft	Divine Healers		Health education
Misconceptions on mode of transmission identified were:	The misconceptions on mode of transmission may result in:	These misconception will lead to:	The interventions to address these misconception are:
Airborne	Negative attitude towards suspected EVD cases	Containment challenges	Health education
Mosquitoes	Conflict of beliefs with health educators	Containment challenges	Health education to demystify the misconceptions
Houseflies		Containment challenges	Health education to demystify the misconceptions

The present study revealed a potential tendency to stigmatize and discriminate against suspected patients of EVD at both home and the health facility. This stigma and discrimination emanates from two main sources: (1) perceived deadly nature of EVD and how it is transmitted, and (2) misconceptions on causes, mode of transmission, and management. Though EVD was perceived to be deadly, the general belief that houseflies and mosquitoes could transmit EVD presents a situation where no individual in a community would like to associate with any suspected cases of EVD. The study revealed a high likelihood of stigmatization and discrimination towards suspected or confirmed EVD patients, as well as members of their households and close associates as has been observed in other infectious conditions [40].

A review of articles on EVD-related stigma concluded that EVD patients suffer stigma that was similar to HIV/AIDS, which often resulted in neglect by close contact [42]. Another study on HIV related misconceptions among Chinese, revealed that participants with a higher level of misconceptions about HIV transmission had stronger discriminatory attitude towards suspected HIV patients [43]. A knowledge, attitude and practice study on EVD in Sierra Leone also reported a high level of stigma and discrimination against EVD patients and their households [11]. This stigmatization and fear of isolation of patients in the health care system may also result in some households unwilling to send suspected EVD cases to hospital as has been identified in this study. At the community level, stigma could also lead to patient neglect and denial of care. In Liberia, it was reported that infected people and their households were marginalised by their own neighbourhoods making it difficult for people who have successfully recovered from EVD to reintegrate into the community after being quarantined [44]. The stigma is also closely related to the misconceptions on the modes of transmission of EVD. It is also possible that the fear-provoking messages that dominate the behavioral change communication strategies may be fueling stigmatization [33,34,45]. This will require good health education to disabuse these myths. Behavioral change communication messages must be designed to pass on correct information without necessarily sensationalizing the situation through fear-provoking messages.

The study further showed that EVD-related stigma could also extend to the health care workers who agree to take care of EVD patients as well as their family members. With the likelihood of high stigma against health care workers who agree to work in EVD treatment centres, many health workers may be unwilling to accept postings to these centres. Lessons from HIV should guide the need to refocus and direct messages towards misconceptions as misconception-related stigma and discrimination have been reported as a major barrier in control and prevention of HIV [46–48]. Refusal of health workers to work in EVD centres could have serious implication in handling and containing EVD should there be an outbreak.

The levels of stigma, its effects on EVD control and prevention as derived from the study have been summarized with strategic points to assist in dealing barriers to behaviors in Table 4.

**Table 4:** EVD-related stigma, effects and interventions.

Level of stigma	Effects of stigma	Strategies
**Community level**		
Community level stigma will be directed as:	The community level stigma will result in:	The strategies to be employed include:
EVD patients	Patients may be abandoned	Targeted health education
Household members	Hiding of patients	Involving community leaders
Compound members	Post-treatment stigma	
Relatives	Denial of condition	
**Health system-related stigma**		
The health system stigma will be directed at:	The health system related stigma may lead to:	The strategies to use to reduce stigma will include:
EVD patients	Neglect of patients	Training of health care providers
Health care workers in EVD treatment centres	Refusal to work in EVD treatment centres	Provision of PPE
Relatives of health care workers	Lack of confidence in health system	Health education

### Limitations of the Study

The authors used independent language experts to do the translations from local languages to English and these translations were verified. However, it is possible some of the original words could have lost their meaning through the translation process. To mitigate this weakness, in determining the themes, emphasis was placed on overarching themes present in the transcripts rather than specific words or phrases used by respondents. The study was also conducted in only five of the ten regions in Ghana and this has potential effects on how to generalize the findings of this to regions that did not take part in the study. However, the selection of the regions were conducted in such a way to reflect the main the ethnic groupings in Ghana. Sampling technique were used to recruit both community members and health professionals who provide health care to community members. Though the number in each category may be small, the study provided relevant information that will contribute to the design of the information, education and communication strategies in the country. The use of the qualitative approach made it possible to collect in-depth information and social norms in the community which can then be used to design a questionnaire for any quantitative study.

## Conclusions

Knowledge on the causes, modes of transmission, signs and symptoms of EVD is high among Ghanaians who participated in the study, however, many hold misconceptions about the causes of EVD, and modes of transmission. The high EVD-related misconception could lead to intense fear. Also, these misconceptions could lead to stigma and discrimination against suspected EVD cases and their household members. Therefore, public health education should go beyond creating awareness on EVD to targeting these misconceptions as these could emerge as a challenge to EVD containment in Ghana.
